# Effect of Anthropogenic Aerosol Addition on Phytoplankton Growth in Coastal Waters: Role of Enhanced Phosphorus Bioavailability

**DOI:** 10.3389/fmicb.2022.915255

**Published:** 2022-06-17

**Authors:** Qin Wang, Chao Zhang, Haoyu Jin, Ying Chen, Xiaohong Yao, Huiwang Gao

**Affiliations:** ^1^Key Laboratory of Marine Environment and Ecology, Frontiers Science Center for Deep Ocean Multispheres and Earth System, Ocean University of China, Ministry of Education of China, Qingdao, China; ^2^Laboratory for Marine Ecology and Environmental Sciences, Pilot National Laboratory for Marine Science and Technology, Qingdao, China; ^3^Shanghai Key Laboratory of Atmospheric Particle Pollution Prevention, Department of Environmental Science and Engineering, Fudan University, Ministry of Education of China, Shanghai, China

**Keywords:** atmospheric deposition, nutrients, phytoplankton, size structure, China coastal waters, alkaline phosphatase

## Abstract

Atmospheric deposition can supply nutrients to induce varying responses of phytoplankton of different sizes in the upper ocean. Here, we collected surface and subsurface chlorophyll *a* maximum (SCM) seawaters from the Yellow Sea and East China Sea to conduct a series of onboard incubation experiments, aiming to explore the impact of anthropogenic aerosol (AR, sampled in Qingdao, a coastal city in Northern China) addition on phytoplankton growth using schemes with (unfiltered seawater, UFS) and without (filtered seawater, FS) microsized (20–200 μm) cells. We found that AR addition stimulated phytoplankton growth obviously, as indicated by chlorophyll *a* (Chl *a*) in surface incubations, and had stimulatory or no effects in SCM incubations, which was related to nutrient statuses in seawater. The high ratio of nitrogen (N) to phosphorus (P) in the AR treatments demonstrated that P became the primary limiting nutrient. The alkaline phosphatase activity (APA), which can reflect the rate at which dissolved organic P (DOP) is converted into dissolved inorganic P, was 1.3–75.5 times higher in the AR treatments than in the control, suggesting that AR addition increased P bioavailability in the incubated seawater. Dinoflagellates with the capacity to utilize DOP showed the dominant growth in the AR treatments, corresponding to the shift in phytoplankton size structure toward larger cells. Surprisingly, we found that nanosized (2–20 μm) and picosized (0.2–2 μm) Chl *a* concentrations in UFS were generally higher than those in FS. The APA in UFS was at least 1.6 times higher than in FS and was proportional to the contribution of microsized cells to the total Chl *a*, suggesting that microsized cells play an important role in the increase in APA, which contributes to the growth of nanosized and picosized phytoplankton. Current work provides new insight into the increase of P bioavailability induced by atmospheric deposition and resultant ecological effect in coastal waters.

## Introduction

Atmospheric deposition can supply a considerable amount of nutrients, including macronutrients such as nitrogen (N) and phosphorus (P), and micronutrients such as iron (Fe) and zinc (Zn), to the ocean (Jickells et al., 2005; Hooper et al., 2019), and affect the phytoplankton size structure and community composition (Okin et al., 2011; Marañón et al., 2015; Zhang et al., 2019). The deposition of a large amount of N promoted the growth of diatoms and inhibited the growth of diazotrophs in the Bay of Bengal and the Arabian Sea (Krishnamurthy et al., 2007). Dust additions increased the N:P ratios in the seawater and induced the dominant growth of nanosized (2–20 μm) phytoplankton in the East China Sea (ECS) (Zhang et al., 2019). The shift in phytoplankton size structure is always accompanied by the competition for nutrients among phytoplankton of different sizes (Stolte and Riegman, 1996; Hutchins et al., 1999). Large phytoplankton (≥2 μm in cell size) have a greater capacity for biomass accumulation and anti-predator defense (Agusti and Kalff, 1989; Finkel, 2007), leading to an advantageous growth in eutrophic seawater. With minimal diffusion boundary layer thickness and a larger specific surface area (Pasciak and Gavis, 1974; Finkel, 2007; Marañón, 2015; Wei et al., 2019), picosized (0.2–2 μm in cell size) phytoplankton have a competitive advantage in oligotrophic seawater (Chisholm, 1992; Finkel, 2007). However, this consensus is roughly defined, and the nutrient competition mechanism between phytoplankton of different sizes is rather complicated in realistic conditions, where the trophic status is not ideally eutrophic or oligotrophic (Chisholm, 1992; Finkel, 2007). For example, in contrast to diatoms, dinoflagellates have a growth advantage in high-nitrate and low-phosphate seawaters due to their acclimatization to high ratios of N:P (Moore et al., 2013; Zhang et al., 2019), even if there is an overlap in the size structure of diatoms and dinoflagellates. In high nutrient low chlorophyll (HNLC) and coastal seawaters, dust additions can induce the rapid growth of different kinds of diatoms covering nanosized and microsized cells (Boyd et al., 2007; Zhang et al., 2018). Our quantitative knowledge of the relationship between nutrient uptake and the growth of different sized phytoplankton is still inadequate.

The impact of atmospheric deposition on primary productivity is generally associated with the substantial supply of N and/or Fe nutrients, whereas few studies focus on P due to its negligible supply relative to N and Fe (Okin et al., 2011; Kim et al., 2014; Wu et al., 2018). Although some studies pointed out that atmospheric deposition can promote the utilization of DOP to relieve P limitation by providing cofactors such as Fe and Zn in open oceans primarily characterized by oligotrophy (Mahaffey et al., 2014; Browning et al., 2017), there are few studies reported in coastal waters characterized by mesotrophy and even eutrophy. In the context of the overwhelming input of N relative to P through various ways such as riverine input and atmospheric deposition, the phenomenon of P limitation becomes increasingly prevailing in coastal waters (Zheng and Zhai, 2021). The impact of atmospheric deposition on marine phytoplankton is not only confined to the traditional relationship between supply (e.g., N and Fe supply) and demand (e.g., N and Fe limitation), but also considering the acclimatization mechanism to copy with the potential P deficiency. A few studies have deduced that atmospheric deposition might enhance the utilization of DOP in P-deficient environments by calculating the P budget in the system and setting up model parameters (Chu et al., 2018; Zhang et al., 2018). However, there is still a lack of direct evidence to verify this hypothesis in coastal waters, and the resultant ecological effect is still poorly understood.

The Yellow Sea (YS) and ECS adjacent to the East Asian continent are marginal seas of the northwestern Pacific Ocean and are obviously influenced by anthropogenic air pollutants from the surrounding continent (Wang et al., 2000; Zhang and Gao, 2007). A series of studies reported that anthropogenic aerosols can transport a long distance to reach coastal seas and even open oceans (Fu et al., 2015; Kang et al., 2017; Xiao et al., 2018). The source apportionment results also showed that particles collected in the YS were full of secondary, biomass burning, and soot-like particles, indicating that marine aerosols are strongly affected by anthropogenic activities (Du et al., 2012; Fu et al., 2015; An et al., 2019). The N:P ratio in anthropogenic aerosol (AR) is generally much higher than the phytoplankton stoichiometry (i.e., Redfield ratio: N:P = 16:1). In the Jiaozhou Bay of the YS, the N:P ratio of atmospheric dry deposition is higher than 100 and even exceeds 1,000 in some specific conditions (Xing et al., 2017; Wu et al., 2018). It has been reported that anthropogenic N deposition has the potential to change nutrient structure in the seawater (Kim et al., 2014). Atmospheric N deposition is regarded as an important factor that induces phytoplankton blooms (Tan and Shi, 2012; Tan and Wang, 2014). On the other hand, under the impact of vertical water mixing, the nutrients in atmospheric deposition can be transferred to the subsurface layers. Model studies have shown that the supplementation of N in surface waters to the lower layer is an important reason for the formation of subsurface chlorophyll *a* maximum (SCM) (Hodges and Rudnick, 2004; Gong et al., 2017). In contrast to the surface layer, few studies focused on the impact of atmospheric deposition on phytoplankton in the SCM layer.

In this study, we carried out three onboard incubation experiments enriched with AR using surface and SCM seawaters in the YS and ECS. The unfiltered and filtered (through 20-μm membrane) seawaters were used to illustrate the effects of microsized (20–200 μm) phytoplankton on the growth and nutrient uptake of nanosized (2–20 μm) and picosized ones. Based on this, our study intended to (1) reveal the difference in phytoplankton response to AR addition in surface and SCM seawaters; (2) identify the main factor of AR addition that affects the growth and community structure succession of phytoplankton; and (3) explore the interaction between different sized phytoplankton under the effects of AR addition.

## Materials and Methods

### Incubation Experiments

The AR samples used for incubation experiments were collected with a cellulose acetate filter membrane (Whatman 41) on the Laoshan campus of Ocean University of China (36°9’39”N, 120°29’29”E) on 30 June 2019. During the sampling period, the AQI was 57–83 μg m^–3^, indicating that the air quality is moderate. The detailed air quality conditions are shown in Table 1.

**TABLE 1:** Air quality conditions during the anthropogenic aerosol (AR) sampling period.

Parameter	Concentration
Humidity (%)	62–91
AQI (μg⋅m^–3^)	57–83
PM_2.5_ (μg⋅m^–3^)	29–61
PM_10_ (μg⋅m^–3^)	63–94
NH_4_^+^ (μmol⋅m^–3^)	0.17
NO_3_^–^ + NO_2_^–^ (μmol⋅m^–3^)	0.43
PO_4_^3–^ (nmol⋅m^–3^)	7.37
Fe (nmol⋅m^–3^)	31.88
Zn (nmol⋅m^–3^)	2.31
Al (nmol⋅m^–3^)	72.24
Mn (nmol⋅m^–3^)	0.98
Cu (nmol⋅m^–3^)	0.19
Cd (nmol⋅m^–3^)	0.01
Ni (nmol⋅m^–3^)	0.13
Pb (nmol⋅m^–3^)	0.13
Co (nmol⋅m^–3^)	0.01

*Humidity data during sampling were obtained from National Meteorological Information Center (NMIC, https://data.cma.cn/). AQI, PM_2.5_, and PM_10_, were obtained from China National Environmental Monitoring Center (CNEMC, http://www.cnemc.cn/). The soluble nutrients and total trace metal concentrations in aerosols were sampled and determined in the laboratory (Zhao et al., 2015).*

**FIGURE 1:**
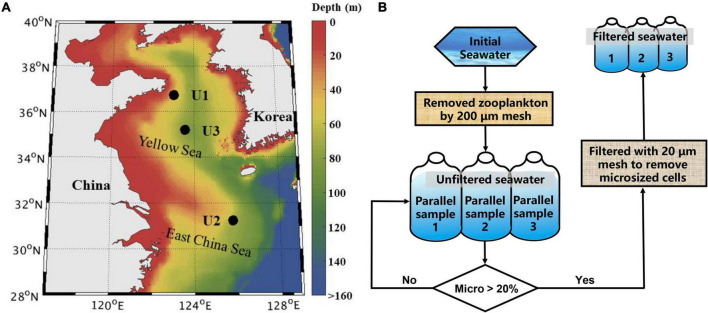
Water sampling stations used for the microcosm incubation experiments in the Yellow Sea and East China Sea **(A)** and the treatment procedure of the incubations **(B)**. Where micro refers to the contribution of microsized Chl *a* to total Chl *a*.

In the summer of 2019, three onboard microcosm experiments were conducted during the cruise of R/V Beidou in the YS and the northern part of the ECS (Figure 1A). The initial seawater at U1, U2 and U3 was collected from surface layers (∼3–5 m below the water surface) and SCM layers (captured by the CTD profile data) using Niskin bottles with Sea Bird CTD-General Oceanic Rosette assembly (Table 2). The site name is abbreviated as Ui_*Sur*_ and Ui_*SCM*_, respectively, and i refers to the site number, i.e., 1/2/3.

**TABLE 2:** Background conditions of seawater at the experimental sites.

Site	U1		U2		U3	
Incubation dates (2019)	Aug 16-21		Sep 3-8		Aug 22-28	
Water layer	Surface	SCM	Surface	SCM	Surface	SCM
Water depth (m)	3	19	3	34	3	29
Temperature (°C)	24.2	18.0	28.6	26.7	27.7	20.3
Salinity	31.5	31.8	31.4	32.8	30.2	32.6
NO_3_^–^ + NO_2_^–^ (μmol⋅L^–1^)	0.19	1.70	0.08	2.59	0.10	0.11
PO_4_^3–^ (μmol⋅L^–1^)	0.01	0.17	ND	0.11	0.01	0.01
Si(OH)_4_ (μmol⋅L^–1^)	2.85	4.78	1.34	4.90	1.71	0.98
N:P (μmol:μmol)	15:1	10:1	20:1	24:1	12:1	11:1
APA (nmol⋅(L⋅h)^–1^)	35.3	3.4	2.2	3.7	18.4	4.7
Chl *a* (μg⋅L^–1^)	0.38	1.19	0.55	0.95	0.62	0.58
Micro Chl *a* (%)	9	26	8	8	9	15
Nano Chl *a* (%)	18	22	27	30	36	38
Pico Chl *a* (%)	73	52	65	62	55	47
Dinophyceae (%)	60	95	85	89	92	96

*ND, not detectable.*

Based on the content of N in AR aerosols, three treatments were conducted in triplicate for the incubation experiments: (1) control, no AR addition; (2) low AR addition, the added amount of AR was expressed in the unit of N (i.e., 1 μmol N L^–1^) at U2, indicating that the added amount of AR contains 1 μmol N L^–1^; (3) high AR addition, 1.7 μmol N L^–1^at U3, and 2 μmol N L^–1^ at U1. AR sample was first ultrasonically extracted in deionized water at 0°C for 1 h, and the leaching solution including particles was added to the incubation bottles directly (Guo et al., 2013). Apart from inorganic nutrients, the aerosol additions could stimulate mixotrophic dinoflagellates by promoting the utilization of organic matter (Granéli et al., 1999; Heisler et al., 2008; Lin et al., 2012). With N addition of 1 μmol L^–1^, 1.7 μmol L^–1^, and 2 μmol L^–1^ by AR particles, the P concentrations added by AR particles were only 17.98, 30.56, and 35.95 nmol L^–1^. The added amount of AR was determined according to the deposition flux of N (574.0–970.0 mg m^–2^ event^–1^) divided by an averaged mixed layer of 30 m (Shi et al., 2012; Sin et al., 2013). Nutrient enrichment experiments were set up to illustrate nutrient limitation in original seawaters and interpret phytoplankton response to AR additions (Supplementary Table 1).

The sampled seawater was passed through 200-μm sieves, mixed well in a clean 120-L high-density polyethylene barrel, and then dispensed into clean (acid-washed) 20-L polycarbonate incubation bottles. These incubation bottles were placed into three microcosm devices filled with continually updated surface seawater to keep the incubation system temperature relatively stable (Zhang et al., 2019). To explore the impact of microsized cells on the phytoplankton community, seawater from incubation bottles of each treatment was filtered through 20-μm sieves when the contribution of microsized Chl *a* to total Chl *a* in the control exceeded 20%, and then, the filtered seawater (hereafter FS) was transferred uniformly into three 2-L bottles to continue the cultivation. The incubations unfiltered with 20-μm sieves were defined as the unfiltered seawater (hereafter UFS, Figure 1B). Bottles were shaded to have approximately 40% light attenuation, matching light levels at depths on 3–5 m as previously used (Zhang et al., 2019, 2020). All experiments ran for 5–6 days.

### Measurements of Chlorophyll *a*, Nutrients, and the Phytoplankton Community Structure

#### Chlorophyll *a*

Approximately 150 ml of seawater from incubated bottles was sampled at ∼07:00 a.m. every day during the incubations. The sampled seawater was subsequently filtered through 20-, 2-, and 0.2-μm filters, to obtain microsized, nanosized, and picosized cells. After 20–24 h of extraction by 90% acetone in darkness at –20°C, the pigments collected by different filters were measured using a Trilogy fluorometer (Turner Designs). The total Chl *a* concentration was obtained by summing three size-fractionated Chl *a* concentrations.

#### Nutrients

An ultrasonic method was used to leach nutrients in AR samples. Briefly, AR samples were ultrasonically extracted in deionized water at 0°C for 1 h. The leaching solution was then filtered through a 0.45-μm polyethersulfone syringe filter (Shi et al., 2010). The filtrates were used for the determination of soluble nutrients from aerosols, including NO_3_^–^, NO_2_^–^, NH_4_^+^, Si(OH)_4_, and PO_4_^3–^. In addition, ∼200 ml of incubated seawater (sampled every day) was filtered through acid-washed cellulose acetate membranes into 125-ml acid-washed high-density polyethylene bottles (prerinsed with the filtrates three times). The water samples were frozen at -20°C immediately prior to the determination of NO_3_^–^ + NO_2_^–^, PO_4_^3–^, and Si(OH)_4_ in the university laboratory. All nutrient samples were measured with a QuAAtro continuous-flow analyzer (SEAL Analytical). The detection limits for NH_4_^+^, NO_3_^–^,NO_2_^–^, PO_4_^3–^, and Si(OH)_4_ were 0.04, 0.02, 0.005, 0.01, and 0.03 μmol L^–1^, respectively. For convenience, NO_3_^–^ + NO_2_^–^ is abbreviated to N + N.

The concentrations of total trace metals were analyzed by inductively coupled plasma mass spectrometry (ICP-MS) (Shi et al., 2012). The 8-cm^2^ cellulose acetate filter was put into the Teflon high pressure vial, with 2 ml of 69% HNO_3_ and 0.5 ml of 40% HF. After digestion at 180°C for 48 h, the solution was evaporated at 160°C, the residue was dissolved with 2% HNO_3_ and diluted to 50 ml for determination.

#### Alkaline Phosphatase Activity

About 45 ml of seawater was sampled from the incubated bottles and mixed with 0.5 ml of fluorogenic substrate 4-methylumbelliferone phosphate (MUF-P) as the mixed substrate. After the addition of the mixed borax-sodium carbonate buffer solution (pH = ∼11) and the mixed substrate to the sample tube, the fluorogenic substrate MUF-P hydrolyzed by AP was converted into equimolar phosphate group and 4-methylumbelliferone (MUF). The fluorescence value of MUF was measured and recorded by a Trilogy fluorometer (Turner Designs) at 0, 0.5, and 1 h (Sebastián et al., 2004). The slope was calculated as the hydrolysis rate, which reflect the alkaline phosphatase activity (APA).

#### High-Throughput Sequencing

Approximately, 1 L of seawater from incubated bottles was filtered using 0.22-μm Whatman polycarbonate filters under gentle vacuum pressure (≤0.02 MPa). Filters were stored immediately in liquid nitrogen until DNA extraction and high-throughput sequencing (Shanghai Personal Biotechnology Co., Ltd., Shanghai, China). The V4 hypervariable region was selected as the target region of the 18S rDNA (Liu et al., 2021). The primers for polymerase chain reaction (PCR) were forward primer 582F, 5’-CCAGCASCYGCGGTAATTCC-3’ and reverse primer V4R, 5’-ACTTTCGTTCTTGATYRA-3’ (Hernández-Ruiz et al., 2020). The Illumina NovaSeqPE250 platform was used for paired-end sequencing of community DNA fragments. First, we demultiplexed the raw sequence data and then invoked QIIME cutadapt trim-paired to cut the primers (Martin, 2011). Quality control of these sequences was performed using the DADA2 plugin with QIIME dada2 denoise-paired (Callahan et al., 2016). Then, we merged amplicon sequence variants (ASVs) and removed singleton ASVs. A pretrained naive Bayes classifier plugin was used to annotate the species for each ASV using QIIME2 software (2019.4) (Bokulich et al., 2018). The SILVA database (Release132)^1^ (Quast et al., 2013) was used for species annotation. The microbiome bioinformatics of communities was analyzed using QIIME2 (2019.4). The accession number in NCBI Sequence Read Archive was PRJNA835313.

### Data Analysis

We used one-way ANOVA to assess whether there was a significant difference in the Chl *a* concentration between the control and treatments (Andersen et al., 2020) and evaluated the nutrient limitation in surface seawater at the three sites. Statistical analysis was performed using IBM SPSS Statistics 20 (SPSS 20.0). CANOCO software (version 5.0) was used to analyze the relationships between environmental factors and phytoplankton. The detrended correspondence analysis (DCA) used species-sample data showed that the first axis of gradient was less than 3. Therefore, redundancy analysis (RDA) was the better choice.

## Results

### Overview of Original Seawater in Surface and Subsurface Chlorophyll *a* Maximum Layers

In general, trophic statuses in surface seawater at U1_*Sur*_, U2_*Sur*_, and U3_*Sur*_ and in SCM seawater at U3_*SCM*_ were lower than those in SCM seawater at U1_*SCM*_ and U2_*SCM*_. Phytoplankton at U1-3_*Sur*_ were colimited by N and P based on the significant increase in Chl *a* after N + P addition relative to the control treatment (on days 4–6, *p* < 0.05, Supplementary Figure 1).

At U1_*Sur*_, the concentrations of N + N, PO_4_^3–^, and Si(OH)_4_ were 0.19, 0.01, and 2.85 μmol L^–1^, respectively. APA was 35.3 nmol (L h)^–1^ in the original seawater, consistent with the reported value of ∼35 nmol (L h)^–1^ in this region (Wang et al., 2014). The total Chl *a* concentration was as low as 0.38 μg L^–1^, of which picosized cells (73%) contributed to the most of the total Chl *a*, followed by nanosized cells (18%) and microsized cells (9%) (Table 2). Chloropicophyceae and Dinophyceae codominated the community, with a relative abundance of 33 and 60%, respectively.

At U2_*Sur*_, U3_*Sur*_ and U3_SCM_, low concentrations of N + N and PO_4_^3–^ were observed, i.e., 0.08-0.11 and ∼0.01 μmol L^–1^, respectively. The APA was less than 18.4 nmol (L h)^–1^, and the total Chl *a* concentration (0.55-0.62 μg L^–1^) was more than 1.4 times higher than that at U1_*Sur*_. Picosized Chl *a* contributed to the most of the total Chl *a* (47-65%), and the contribution of microsized Chl *a* was less than 15%. Dinophyceae dominated the communities with a relative abundance of ≥85% (Table 2).

Seawaters at U1_*SCM*_ and U2_*SCM*_ contained abundant nutrients, with 1.70-2.59 μmol L^–1^ of N + N, 0.11-0.17 μmol L^–1^ of PO_4_^3–^, and 4.78-4.90 μmol L^–1^ of Si(OH)_4_. The APA at U1_*SCM*_ and U2_*SCM*_ [3.4-3.7 nmol (L h)^–1^] was of the same orders of magnitude as those of U2_*Sur*_ and U3_*SCM*_. The total Chl *a* concentration (≥0.95 μg L^–1^) was more than 1.7 times higher than that of U1_*Sur*_ and U2_*Sur*_. Picosized phytoplankton (52-62%) were the primary contributors to the total Chl *a*. Dinophyceae dominated the communities with a relative abundance of ≥89% (Table 2).

### Changes in Inorganic Nutrients

The N:P ratio after AR addition increased from 11:1-20:1 to 44:1-54:1 at all sites in the surface incubated seawater and at U3_*SCM*_ in the SCM incubated seawater. Due to the sufficient nutrient stock in the original seawater at U1_*SCM*_ and U2_*SCM*_, the N supplied by AR addition only increased the N:P ratio from 10:1-24:1 to 16:1-28:1. Because of the low contents of PO_4_^3–^ and Si(OH)_4_ in the AR, the changes in the concentrations of PO_4_^3–^ and Si(OH)_4_ in AR-amended seawater were slight at all sites.

**FIGURE 2:**
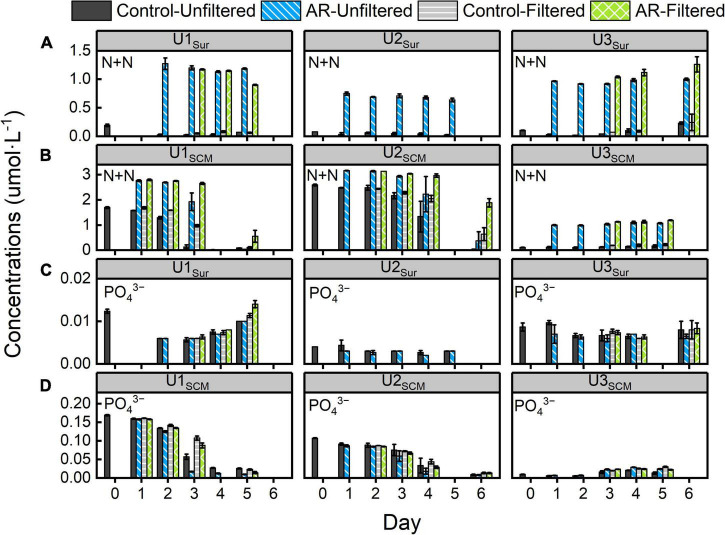
Changes in panels **(A,B)** N + N and **(C,D)** PO_4_^3–^ concentrations in the control and anthropogenic aerosol (AR) treatments incubated with surface and subsurface chlorophyll *a* maximum (SCM) seawaters. The error bar represents the standard deviation of three parallel samples.

During the incubations at U1-3_*Sur*_ and U3_*SCM*_, the concentrations of N + N did not change significantly in the control and AR treatments (Figure 2). In the AR treatments, the N + N concentrations remained relatively stable and were significantly higher than those in the control treatments. In contrast, the concentrations of PO_4_^3–^ were close to the detection limit in all treatments. The maximum consumption of Si(OH)_4_ in the control and AR treatments was less than 13% at U1-3_*Sur*_ and U3_*SCM*_. At U1_*SCM*_ and U2_*SCM*_, the concentrations of N + N and PO_4_^3–^ decreased gradually by more than 85% in the control and AR treatments relative to the original values. The concentrations of Si(OH)_4_ decreased sharply in the control (>28%) and AR treatments (>69%) on days 3-5 at both sites (Figure 2 and Supplementary Figure 2).

There were no obvious differences in the concentrations of N + N, PO_4_^3–^, and Si(OH)_4_ between FS and UFS in the control and AR treatments at U1-3_*Sur*_ and U3_*SCM*_. In the FS at U1_*SCM*_ and U2_*SCM*_, the consumption of N + N and Si(OH)_4_ in AR treatments was lower than that in the UFS at the end of the incubations (*p* < 0.05, Figure 2 and Supplementary Figure 2).

### Changes in Alkaline Phosphatase Activity

**FIGURE 3:**
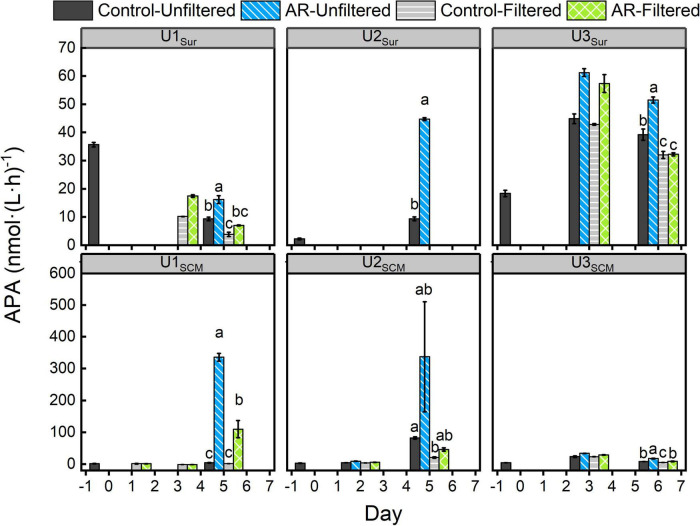
Changes in alkaline phosphatase activity (APA) in the control and AR treatments incubated with surface and SCM seawaters. Treatment in the last day was compared by Ducon’s range test, and the treatment with same letter was not significantly different (α = 0.05). The error bar represents the standard deviation of three parallel samples.

In the UFS, the APA in the AR treatments was 1.3-75.5 times higher than that in the control at the end of the incubations (*p* < 0.05, Figure 3), and this phenomenon could be observed in both surface and SCM incubations. In contrast, there was almost no difference in APA between control and AR treatments on the last day of FS incubations at all sites except U1_*SCM*_. For the AR treatments, the APA in the UFS at all sites was generally higher (1.6-7.3 times) than that in the FS (Figure 3).

### Changes in Total and Size-Fractionated Chlorophyll *a*

**FIGURE 4:**
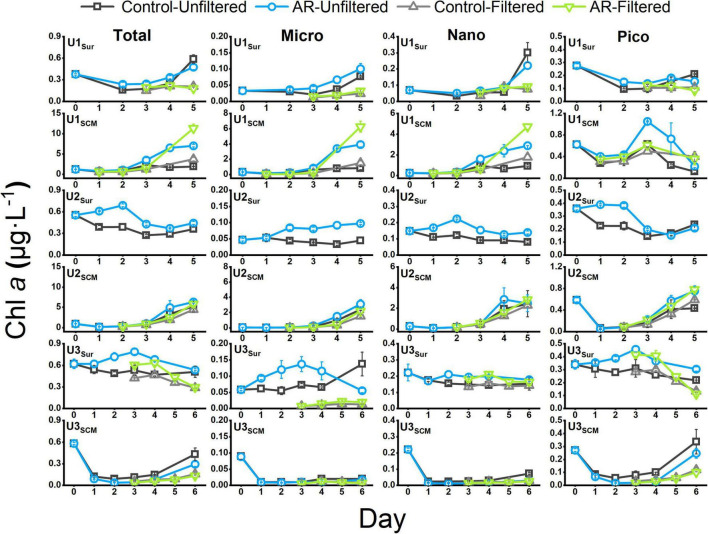
The concentration of Chl *a* in the control and AR treatments incubated with surface and SCM seawaters. The error bar represents the standard deviation of three parallel samples.

**FIGURE 5:**
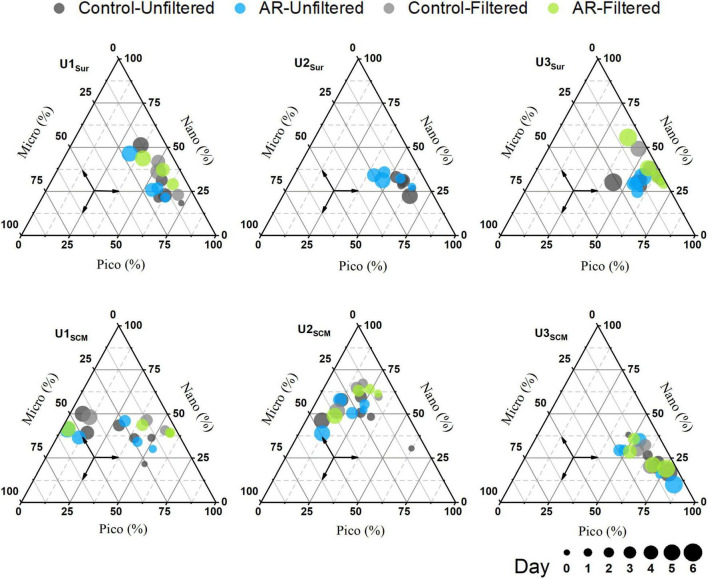
Changes in size structure of phytoplankton in the control and AR treatments incubated with surface and SCM seawaters.

At U1-3_*Sur*_, the concentration of the total Chl *a* in the AR treatments was generally higher than that in the control. At U1_*SCM*_, the total Chl *a* concentration in the AR treatments was more than 1.2 times higher than that in the control on days 2-5. At U2_*SCM*_ and U3_*SCM*_, there was no significant difference in Chl *a* between the control and AR treatments (Figure 4). The responses of phytoplankton of different sizes varied with AR addition. At U1_*Sur*_, the dominant size of phytoplankton changed from picosize to nanosize (46% contribution to total Chl *a*). A similar pattern in the size shift toward larger cells also occurred at U2_*Sur*_ and U3_*Sur*_, although picosized cells always dominated the contribution to total Chl *a* (Figure 5). At U1_*SCM*_ and U2_*SCM*_, the dominant contributor of phytoplankton in AR treatments was picosized cells on days 1-2 and shifted to microsized cells (≥49% contribution to total Chl *a*) on day 5. At U3_*SCM*_, the dominant contributor was always picosized phytoplankton during the incubations (Figure 5).

For AR treatments, the concentrations of nanosized and picosized Chl *a* in UFS were generally higher than those in FS at U1_*Sur*_ and U3_*Sur*_. Specifically, at U1_*Sur*_, nanosized and picosized Chl *a* concentrations in the UFS enriched with AR were 2.4 and 1.8 times higher than those in the FS on day 5. At U3_*Sur*_, the picosized Chl *a* concentration in UFS enriched with AR was 2.7 times higher than that in FS on day 6. Similar to the incubations with surface seawater, the picosized Chl *a* concentration enriched with AR in FS (0.10 μg L^–1^) at U3_*SCM*_ was lower than that in UFS (0.25 μg L^–1^) on day 6. In contrast, at U1_*SCM*_, nanosized and picosized Chl *a* concentrations in FS (4.72 and 0.36 μg L^–1^) were higher than those in UFS (2.89 and 0.22 μg L^–1^) on day 5. At U2_*SCM*_, there was no significant difference in the concentrations of size-fractionated Chl *a* between UFS and FS (Figure 4).

### Changes in the Phytoplankton Community

**FIGURE 6:**
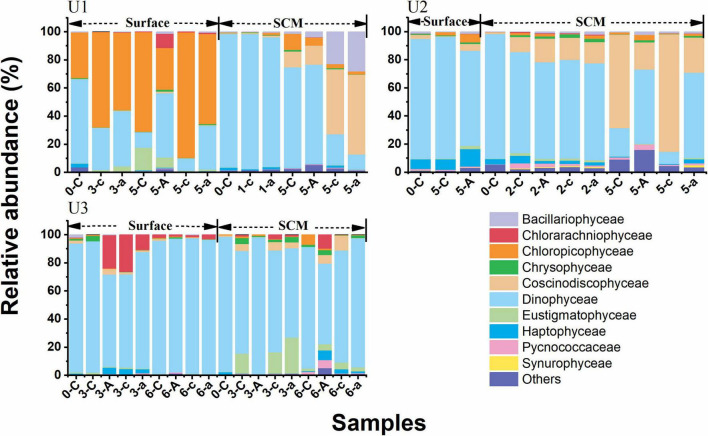
Relative abundances of dominant eukaryotic phytoplankton classes (level 3 of the taxonomic hierarchy in SILVA 132) in the control and AR treatments during the incubation experiments; -C, -A, and -c, -a refer to the control, and AR treatments in the unfiltered and filtered seawaters, e.g., 0-C means on day 0 in the unfiltered seawater for the control treatment. “Others” refers to the groups outside of the top 10.

The ASVs at all sites assigned to phytoplankton could be classified into 25 groups of eukaryotic microalgae at class level (level 3). The ASV richness of Dinophyceae (dinoflagellates) accounted for ≥60% of phytoplankton in the original seawater at each site. In terms of UFS, Dinophyceae dominated the phytoplankton community in the AR treatments at all sites (Figure 6). The dominant class changed from Dinophyceae to Chloropicophyceae (71%) in the control on day 5 of the incubations (corresponding to the maximum Chl *a* concentration) at U1_*Sur*_. *Chloropicon* spp. was the main component of Chloropicophyceae (Supplementary Figure 3). With AR addition, the relative abundance of Dinophyceae increased to 46% being the dominant phytoplankton (Figure 6). The succession of phytoplankton communities at U3_*SCM*_ was similar to that at U1_*Sur*_. Dinophyceae maintained the dominant status in the control and AR treatments at the rest of the incubation sites (Figure 6).

## Discussion

Based on the distinct nutrient concentrations (N, P, and Si) in the original seawaters, the sites used for incubations were classified into two types: U1-3_*Sur*_ and U3_*SCM*_ with lower trophic status, where the concentrations of N + N, PO_4_^3–^, and Si(OH)_4_ did not exceed 0.50, 0.02, or 3.00 μmol L^–1^, respectively; U1_*SCM*_ and U2_*SCM*_ with higher trophic status, where the concentrations of N + N, PO_4_^3–^, and Si(OH)_4_ exceeded 1.50, 0.10, and 4.50 μmol L^–1^, respectively.

### Distinct Responses of Phytoplankton to Anthropogenic Aerosol Additions in Surface and Subsurface Chlorophyll *a* Maximum Seawaters

Aerosol additions generally stimulated phytoplankton growth and shifted the phytoplankton size structure toward larger cells in surface incubated seawaters (Figure 5). This is justified because of the established supplementary relationship between nutrients (primarily N) supplied by AR and phytoplankton requirements. Such fertilization effect has also been widely reported in the previous studies (Cottingham, 1999; Liu et al., 2013; Zhang et al., 2019). In contrast, AR additions had a limited effect on phytoplankton size structure at U3_*SCM*_, although its trophic status was similar to those in surface seawaters (Figure 5). This is ascribed to the photo-acclimation of phytoplankton under the condition of low irradiance in SCM layer (Fujiki and Taguchi, 2002; Fu et al., 2018). With the abrupt enhancement of light intensity (from SCM to surface), phytoplankton in the incubated seawater need to readjust to the new environment and thus showed a limited response to AR additions.

Interestingly, AR addition had a significant fertilization effect on phytoplankton growth at U1_*SCM*_ (Figure 4), which was characterized by the higher trophic status among these sites. Note that there was a shift in dominant phytoplankton from picosized cells to large cells during the incubations at U1_*SCM*_, which was different from the sustaining dominance of picosized cells at U3_*SCM*_ (Figure 5). Moreover, large phytoplankton can better acclimate to the abrupt increase in light intensity compared with picosized cells, due to their stronger self-shading capacity by the pigment (package effect) to reduce light absorption (Marañón, 2015). At U2_*SCM*_, the stimulation effect of AR addition was not as obvious as that at U1_*SCM*_ (Figure 4). This is because there was an obvious shift of dominant algae from Coscinodiscophyceae in the control to Dinophyceae in the AR treatments at U2_*SCM*_ (Figure 6). The obvious succession in phytoplankton community while slight change in Chl *a* under the condition of aerosol enrichment was also observed in eutrophic seawaters of the ECS (Meng et al., 2016). The substantial input of N relative to P supplied by AR addition increased the N:P ratio from 24:1 in the original seawater to 28:1 in the AR treatments at U2_*SCM*_, which was more favorable for the growth of dinoflagellates (Zhang et al., 2019; Table 2). At U1_*SCM*_, in contrast, the N:P ratio ranged between 10:1 and 16:1 in the control and AR treatments, leading to the increase in relative abundance of diatoms (primarily Coscinodiscophyceae) (Zhang et al., 2019). Collectively, in contrast to the consistent phytoplankton response to AR addition in surface seawater, the impact of AR addition in SCM seawater is complicated, which is closely related to nutrient concentration and structure in seawater.

### Utilization of Dissolved Organic P Enhanced by Anthropogenic Aerosol Addition

**FIGURE 7:**
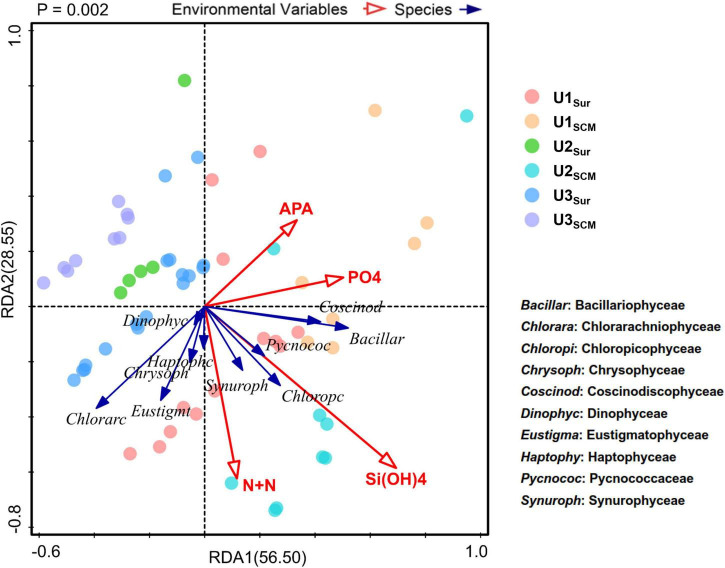
Correlations between species (level 3) and environmental factors based on redundancy analysis (RDA).

The substantial N supplied by AR had the potential to alleviate and even alter N pressure of phytoplankton in the incubated seawater (Figure 2). As a result, the relatively P-deficient environment created by AR additions made it possible for phytoplankton to induce acclimatization mechanisms to copy with P stress (Moore et al., 2013). As shown in Figure 3, the APA value in the AR treatments was higher than that of the control at the end of the incubations at all sites, indicating that AR could enhance the utilization of DOP to increase P bioavailability in the incubated seawater. Such phenomenon was supported by the good correlation between PO_4_^3–^ and APA (Figure 7). In apart from the establishment of P-deficient environment, AR additions also provided a considerable amount of soluble Fe and Zn, which acted as cofactors of phosphohydrolytic enzymes (Mills et al., 2004; Mahaffey et al., 2014). The promotion effect of AR addition on the utilization of DOP shows the acclimation of phytoplankton to the overwhelming N input relative to P in coastal waters (Zhang et al., 1999; Zamora et al., 2010; Xing et al., 2017) and is conductive to understanding P biogeochemical cycles in the perspective of atmospheric deposition.

In terms of the phytoplankton community composition, Dinophyceae generally dominated the community in UFS enriched with AR at all sites (Figure 6). With relatively high tolerance to nutrient-deficient environments and the potential to utilize DOP by inducing the expression of the gene for the synthesis of AP (Lin et al., 2012), dinoflagellates showed an advantageous growth in the AR treatments. This is also the reason why nutrient limitation had a slight impact on the growth of Dinophyceae based on RDA (Figure 7). In addition, due to the selective feeding of micrograzers, picosized phytoplankton suffer from a higher grazing pressure (Cottingham, 1999; Strom et al., 2007). In contrast, large dinoflagellates have the ability to keep themselves away from the prey of the dominant zooplankton species *Paracalanus parvus* (Hexanauplia) through particle rejection behavior (reject particles as food, Supplementary Figure 4; Huntley et al., 1986; Tiselius et al., 2013).

### Role of Microsized Phytoplankton in Affecting the Growth of Nanosized and Picosized Phytoplankton

As described in “Changes in Total and Size-Fractionated chlorophyll *a*” section at U1_*Sur*_, U3_*Sur*_, and U3_*SCM*_ characterized by lower trophic statuses, the nanosized and picosized Chl *a* concentrations in FS enriched with AR were lower than those in UFS. In contrast, we did not observe similar results at U1_*SCM*_ and U2_*SCM*_ characterized by higher trophic statuses (Figure 4).

**FIGURE 8:**
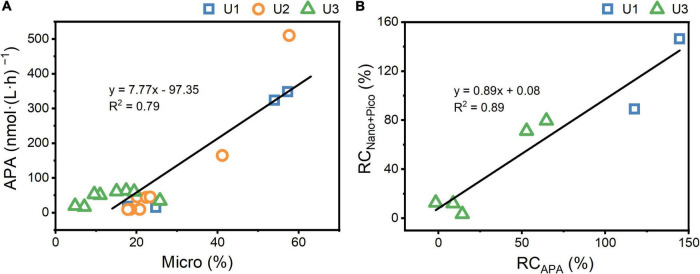
The relationship between APA and the contribution of microsized Chl *a* to the total Chl *a* in the AR treatments **(A)** and the relationship between relative change in APA (RC_*APA*_) and nanosized+picosized Chl *a* (RC_*Nano+Pico*_) in the unfiltered seawater relative to the filtered seawater **(B)**. RC_*APA*_ and RC_*Nano+Pico*_ was calculated as ([mean in the unfiltered seawater - mean in the filtered seawater]/mean in the filtered seawater) × 100. The contribution of microsized Chl *a* to the total Chl *a* in the control was less than 15% throughout the experiments, and thus, there was no incubations with filtered seawater at U2_*Sur*_.

Nutrients, irradiance, and temperature are considered the three major factors that affect phytoplankton growth (Laws et al., 2000; Litchman, 2007). There was no difference in light and temperature between UFS and FS, and thus, nutrients play a key role in causing the lower nanosized and picosized Chl *a* concentrations in FS. At U1-3_*Sur*_ and U3_*SCM*_, there were no obvious differences in N + N and PO_4_^3–^ between UFS and FS (Figure 2). Meanwhile, we found that the APA values in UFS were 1.6-7.3 times higher than those in FS (Figure 3), indicating that microsized cells played an important role in increasing P bioavailability in the incubated seawater (Sebastián et al., 2004). This was supported by the positive linear relationship between APA and the contribution of microsized cells to the total Chl *a* (Figure 8A). As an extracellular enzyme, AP enters the environment through autolyzing or organisms excreting (Štrojsová et al., 2003). Besides, in contrast to FS, nanosized and picosized Chl *a* concentration in UFS increased linearly with relative change of APA (Figure 8B). Therefore, under the impact of AR addition, microsized cells have the ability to favor the growth of nanosized and picosized cells by increasing P bioavailability in seawater. The result at U1_*SCM*_ characterized by higher trophic statuses could also support this argument. On days 1-2 of the incubations, there was no difference in the nanosized and picosized Chl *a* concentrations in the AR treatments between UFS and FS when PO_4_^3–^ was sufficient in the seawater, but lower nanosized and picosized Chl *a* concentrations in FS were measured when PO_4_^3–^ was exhausted on day 3 (Figures 2, 4). Besides, dinoflagellates and green algae with the capacity of utilizing DOP also showed advantageous growth in the P-deficient condition (Figure 6).

There are other factors that might have caused the mismatch between UFS and FS in the concentration of nanosized and picosized Chl *a*. For example, the biodiversity of the community decreased after removal of microsized cells, which increased the difficulty for the community to reestablish a new balance (Dyke et al., 2007). Nanosized and picosized phytoplankton may adopt a strategy to survive in unstable habitats, e.g., by producing spores that can be dormant temporarily and revive at an appropriate time (Nayaka et al., 2017). However, these inferences cannot account for the change in nanosized and picosized Chl *a* at U1_*SCM*_ and U2_*SCM*_ characterized by higher trophic statuses (Figure 4). Therefore, our study provides a new clue from the perspective of nutrient utilization to illustrate how microsized phytoplankton affect the growth of nanosized and picosized ones.

## Conclusion

In this study, we conducted three onboard incubation experiments using surface and SCM seawaters under the condition of sea surface light intensity in the Yellow Sea and East China Sea. AR addition generally stimulated phytoplankton growth in surface incubations and had a stimulatory or slight impact in SCM incubations, which primarily depends on the nutrient concentration and structure in seawater. We also found that AR addition could alleviate P limitation by promoting the utilization of DOP in both surface and SCM incubations. Specifically in seawater with lower trophic status, microsized cells have the ability to promote the growth of nanosized and picosized cells by increasing P bioavailability in the incubated seawater. Considering the lower contribution of microsized cells in the oligotrophic areas of the open oceans (López-Urrutia and Morán, 2015; Marañón et al., 2015), such a promotion effect of microsized cells induced by anthropogenic aerosol deposition may focus on coastal waters and thus can be regarded as a result of anthropogenic influences to a large extent. With the enhanced influence of human activities in the recent years, atmospheric deposition characterized by high N:P ratios has intensified the prevailing P limitation in offshore waters (Harrison et al., 1990; Xu et al., 2008). The acclimation mechanism of different sized phytoplankton to P limitation under the influence of atmospheric deposition deserves to be further investigated.

## Data Availability Statement

The datasets presented in this study can be found in online repositories. The names of the repository/repositories and accession number(s) can be found in the article/Supplementary Material.

## Author Contributions

QW: conceptualization, investigation, methodology, data curation, formal analysis, visualization, software, and writing – original draft. CZ: conceptualization, investigation, writing – review and editing, data curation, and funding acquisition. HJ: investigation. YC and XY: writing – review and editing. HG: supervision, methodology, resources, writing – review and editing, and funding acquisition and also was responsible for ensuring that the descriptions are accurate and agreed by all authors. All authors contributed to the article and approved the submitted version.

## Conflict of Interest

The authors declare that the research was conducted in the absence of any commercial or financial relationships that could be construed as a potential conflict of interest.

## Publisher’s Note

All claims expressed in this article are solely those of the authors and do not necessarily represent those of their affiliated organizations, or those of the publisher, the editors and the reviewers. Any product that may be evaluated in this article, or claim that may be made by its manufacturer, is not guaranteed or endorsed by the publisher.

## Footnotes

1
http://www.arb-silva.de
