# Supplementary material for: Effect of Anthropogenic Aerosol Addition on Phytoplankton Growth in Coastal Waters: Role of Enhanced Phosphorus Bioavailability
Source: Front Microbiol. 2022 Jun 17;13:915255. doi: 10.3389/fmicb.2022.915255 (PMC9247544; doi:10.3389/fmicb.2022.915255)
Supplement: Supplementary file 1 [file Data_Sheet_1.docx]

Supplementary Material

**Table 1**. Protocol of nutrient enrichment for the incubation experiments with surface seawater

**Figure 1**.  Changes in the total Chl *a* concentration in the control, N and N+P treatments with unfiltered seawater during the incubation experiments.

**Figure 2**. Changes in Si(OH)_4_ concentrations in the control and AR treatments incubated with surface and SCM (subsurface chlorophyll *a* maximum) seawater.

**Figure 3**. Relative abundances of dominant eukaryotic phytoplankton species (level 7 of the taxonomic hierarchy in SILVA 132) in the control and AR treatments during the incubation experiments; -C, -A, and -c, -a refer to the control, and AR treatments in the unfiltered and filtered seawaters, e.g., 0-C means day 0 in the control treatment incubated with unfiltered seawater. “Others” refers to the groups outside of the top 22.

**Figure 4**. Relative abundances of dominant species in class Hexanauplia in the control and AR treatments during the incubation experiments; -C, -A, and -c, -a refer to the control, and AR treatments in the unfiltered and filtered seawaters, e.g., 0-C means day 0 in the control treatment incubated with unfiltered seawater. “Others” refers to the groups outside of the top 5.

**Table 1**. Protocol of nutrient enrichment for the incubation experiments with surface seawater

| Sites | Treatments^a^ | Amended concentrations |
| --- | --- | --- |
| U1 | N  N+P | 2 μmol L^-1^  2 μmol L^-1^+0.2 μmol L^-1^ |
| U2 | N | 1 μmol L^-1^ |
|  | N+P | 1 μmol L^-1^+0.2 μmol L^-1^ |
| U3 | N | 1.7 μmol L^-1^ |
|  | N+P | 1.7 μmol L^-1^+0.2 μmol L^-1^ |

^a^ N was provided in the form of NaNO_3_, P was in the form of NaH_2_PO_4_.





**Figure 1**.  Changes in the total Chl *a* concentration in the control, N and N+P treatments with unfiltered seawater during the incubation experiments.





**Figure 2**. Changes in Si(OH)_4_ concentrations in the control and AR treatments incubated with surface and SCM (subsurface chlorophyll *a* maximum) seawater.





**Figure 3**. Relative abundances of dominant eukaryotic phytoplankton species (level 7 of the taxonomic hierarchy in SILVA 132) in the control and AR treatments during the incubation experiments; -C, -A, and -c, -a refer to the control, and AR treatments in the unfiltered and filtered seawaters, e.g., 0-C means day 0 in the control treatment incubated with unfiltered seawater. “Others” refers to the groups outside of the top 22.





**Figure 4**. Relative abundances of dominant species in class Hexanauplia in the control and AR treatments during the incubation experiments; -C, -A, and -c, -a refer to the control, and AR treatments in the unfiltered and filtered seawaters, e.g., 0-C means day 0 in the control treatment incubated with unfiltered seawater. “Others” refers to the groups outside of the top 5.
